# Body mass index and hemophagocytic lymphohistiocytosis: a risk assessment in HIV-positive leukemia patients

**DOI:** 10.1097/MS9.0000000000003195

**Published:** 2025-03-28

**Authors:** Emmanuel Ifeanyi Obeagu, Martin H. Bluth

**Affiliations:** a Department of Biomedical and Laboratory Science, Africa University, Mutare, Zimbabwe; b Department of Pathology, Division of Blood Transfusion Medicine, Maimonides Medical Center, Brooklyn, New York, USA

**Keywords:** body mass index (BMI), HIV, immunosuppression, leukemia, secondary hemophagocytic lymphohistiocytosis (sHLH)

## Abstract

Secondary hemophagocytic lymphohistiocytosis (sHLH) is a severe hyperinflammatory syndrome characterized by excessive activation of the immune system, leading to multiorgan dysfunction and high mortality. This review explores the relationship between body mass index (BMI) and the risk of sHLH in leukemia patients living with human immunodeficiency virus (HIV). In this population, the compounded effects of immunosuppression and inflammatory conditions present unique challenges, making it crucial to understand the role of BMI in modulating these risks. Leukemia and HIV independently contribute to significant immune system dysregulation. When these conditions coexist, patients face increased susceptibility to infections and inflammatory disorders. BMI, an indicator of nutritional and metabolic status, can further influence immune responses. Low BMI, often associated with malnutrition, impairs immune function, while high BMI, linked to obesity, promotes chronic inflammation. Both extremes can exacerbate the cytokine storm characteristic of sHLH, leading to more severe clinical outcomes. Regular monitoring and addressing nutritional status through tailored interventions can potentially mitigate the risk of sHLH. A multidisciplinary approach, integrating the expertise of oncologists, infectious disease specialists, nutritionists, and immunologists, is essential for optimizing patient care and improving outcomes in this vulnerable population. Further research is necessary to deepen our understanding of the mechanisms linking BMI to sHLH and to develop evidence-based guidelines for clinical practice.

## Introduction

Hemophagocytic lymphohistiocytosis (HLH) is a rare but life-threatening condition characterized by excessive immune activation and severe systemic inflammation^[1]^. It can be classified as either primary (familial) HLH, which is usually due to genetic mutations affecting cytotoxic function, or secondary HLH (sHLH), which occurs in response to infections, malignancies, autoimmune diseases, or other triggers. Both forms lead to a cytokine storm, hyperactivation of macrophages and T lymphocytes, and widespread hemophagocytosis in the bone marrow, liver, spleen, and lymph nodes. sHLH is particularly concerning in patients with underlying conditions such as leukemia and human immunodeficiency virus (HIV)^[2]^. Leukemia, a malignancy of the blood-forming tissues, inherently disrupts normal hematopoiesis and immune function. HIV, a retrovirus targeting the immune system, causes chronic immunosuppression and increases susceptibility to opportunistic infections and other immune-related complications^[3,4]^. When leukemia and HIV coexist, the compounded immunosuppressive effects can trigger or exacerbate sHLH. Body mass index (BMI), calculated as weight in kilograms divided by height in meters squared (kg/m^2^), is a key indicator of nutritional and metabolic status^[5]^. It is commonly used to classify individuals as underweight, normal weight, overweight, or obese. BMI influences immune function and inflammation, which are crucial factors in the pathogenesis of sHLH^[6]^.

**HIGHLIGHTS**

- Body mass index (BMI) plays a role in assessing risks in human immunodeficiency virus (HIV)-positive leukemia patients.
- High BMI may worsen immune function and increase inflammation.
- Hemophagocytic lymphohistiocytosis (HLH) exacerbates complications.
- Obesity correlates with higher HLH severity.
- Monitoring BMI AIDS in tailored therapeutic strategies for management.

Low BMI often signifies malnutrition, which can impair the immune response. Malnutrition leads to deficiencies in essential nutrients, weakening the body’s ability to fight infections and mount adequate inflammatory responses. In leukemia patients with HIV, malnutrition-induced immune suppression can increase susceptibility to infections and inflammation, potentially triggering sHLH^[7]^. High BMI, indicative of overweight or obesity, is associated with a state of chronic low-grade inflammation. Adipose tissue, particularly visceral fat, secretes pro-inflammatory cytokines such as tumor necrosis factor-alpha (TNF-α), interleukin-6 (IL-6), and leptin. This persistent inflammatory state can exacerbate the cytokine storm characteristic of sHLH, leading to more severe clinical manifestations and outcomes^[8]^. Both extremes of BMI – low and high – are linked to immune dysregulation^[9]^. Malnourished individuals exhibit reduced T-cell function and altered cytokine production, compromising their ability to control infections and inflammation. Conversely, obese individuals experience hyperactivation of immune pathways, contributing to a heightened inflammatory response. These dysregulated immune responses can predispose leukemia patients with HIV to sHLH. The hallmark of sHLH is a cytokine storm, an uncontrolled release of pro-inflammatory cytokines^[10]^. This storm leads to widespread activation of macrophages and T lymphocytes, resulting in hemophagocytosis and multiorgan damage. In obese individuals, the elevated levels of pro-inflammatory cytokines can further amplify this storm, while in malnourished individuals, the weakened immune response can fail to control the inflammatory cascade, both contributing to the development and severity of sHLH. Recognizing the impact of BMI on sHLH risk is crucial for early identification and management of high-risk patients. Regular monitoring of BMI and nutritional status in leukemia patients with HIV can aid in timely interventions to mitigate sHLH risk. Addressing malnutrition through dietary interventions and supplementation can enhance immune function, while weight management strategies in obese patients can reduce chronic inflammation. Effective management of leukemia patients with HIV at risk of sHLH requires a multidisciplinary approach^[11]^. Collaboration among oncologists, infectious disease specialists, nutritionists, and immunologists is essential for comprehensive care. Personalized treatment plans that consider BMI and nutritional status can improve clinical outcomes by addressing the unique needs of each patient.

## Aim

The aim of this review is to elucidate the complex relationship between BMI and secondary hemophagocytic lymphohistiocytosis (sHLH) in leukemia patients living with HIV.

## Justifications

sHLH is a severe and potentially life-threatening condition characterized by an excessive immune response^[12]^. It is particularly relevant in patients with complex medical conditions such as leukemia and HIV. Both conditions compromise immune function, increasing the risk of sHLH. BMI is a significant factor affecting immune function and inflammatory responses. Both low BMI (indicating malnutrition) and high BMI (indicating obesity) are known to alter immune responses and cytokine profiles, which can influence the development and progression of sHLH. By exploring these relationships, the review aims to fill gaps in knowledge about how BMI-related immune dysregulation contributes to sHLH in the context of leukemia and HIV. Managing leukemia patients with HIV involves addressing multiple challenges, including nutritional deficiencies, metabolic disorders, and immune dysregulation. BMI-related complications add an additional layer of complexity. A detailed review of how BMI affects disease management, diagnosis, and treatment outcomes will help clinicians develop more targeted and effective care strategies for this vulnerable population. Current management strategies for sHLH and its associated conditions may not fully address the impact of BMI on immune dysregulation. Identifying and reviewing effective management strategies tailored to patients with specific BMI-related challenges will aid in developing more precise therapeutic approaches. This includes nutritional interventions, weight management, and integrated care models.

There is a need for further research into the mechanisms linking BMI with immune dysregulation and sHLH. The review aims to identify gaps in current research, propose future directions, and highlight the importance of personalized medicine and innovative therapeutic approaches. This will contribute to advancing the field and improving patient outcomes through targeted research and clinical practice. Effective management of patients with leukemia, HIV, and BMI-related complications requires a multidisciplinary approach. By discussing the roles of various healthcare professionals and the importance of integrated care pathways, the review seeks to promote better coordination and collaboration in patient care, ultimately leading to improved health outcomes. Patients with complex conditions like leukemia and HIV often face disparities in access to care and treatment. By reviewing and emphasizing the importance of tailored management strategies, the review aims to address these disparities and advocate for policies and practices that improve access to necessary resources and interventions.

## Review methodology

### Literature search strategy

The review process began with a comprehensive literature search to gather relevant studies and evidence. The search strategy involved querying several key databases, including PubMed, Scopus, Web of Science, and Embase. The search terms included combinations of keywords such as “Body Mass Index,” “sHLH,” “secondary hemophagocytic lymphohistiocytosis,” “leukemia,” “HIV,” “immune dysregulation,” “malnutrition,” and “obesity.”

Inclusion criteria for selecting studies were:

1. Relevance: Studies must focus on the relationship between BMI and sHLH, specifically in the context of leukemia and HIV.
2. Publication type: Peer-reviewed articles, systematic reviews, clinical trials, and observational studies were included.
3. Language: Only studies published in English were considered.
4. Date range: Studies published within the last 20 years were prioritized to ensure relevance and currency.

Exclusion criteria included non-peer-reviewed sources, studies not focusing on the specific population of interest (i.e. leukemia patients with HIV), and articles not available in full text.

### Data extraction and analysis

Once relevant studies were identified, data extraction was conducted using a standardized form. Key data points included study design, population characteristics, BMI categories (low and high), sHLH outcomes, immune dysregulation mechanisms, and management strategies. Information was systematically organized to facilitate comparison and synthesis.

The extracted data were categorized based on the following themes:

1. Mechanisms linking BMI to immune dysregulation and sHLH: This included understanding how low BMI (malnutrition) and high BMI (obesity) impact immune function, cytokine profiles, and the risk of sHLH.
2. Clinical implications: Analysis of how BMI-related factors influence disease management, diagnosis, and patient outcomes in leukemia and HIV contexts.
3. Management strategies: Review of current strategies for managing malnutrition, obesity, and sHLH, including nutritional support, weight management, and therapeutic interventions.
4. Future directions: Identification of research gaps, emerging therapeutic approaches, and recommendations for improving patient care.

### Synthesis and interpretation

The reviewed studies were analyzed to identify common findings, trends, and discrepancies. A narrative synthesis approach was employed to integrate the results from different studies, highlighting key insights into the relationship between BMI and sHLH. The synthesis focused on:

- Mechanistic insights: Describing how BMI-related factors contribute to immune dysregulation and sHLH.
- Clinical insights: Discussing the impact of BMI on disease management and patient outcomes.
- Strategic insights: Evaluating current management practices and proposing potential improvements.

### Quality assessment

The quality of the included studies was assessed using established criteria, such as the Cochrane Risk of Bias Tool for randomized controlled trials and the Newcastle-Ottawa Scale for observational studies. This assessment helped ensure that the review findings were based on high-quality evidence.

### Understanding sHLH

sHLH is a hyperinflammatory syndrome resulting from an excessive and uncontrolled immune response^[13]^. Unlike primary HLH, which is driven by genetic mutations, sHLH occurs due to external triggers such as infections, malignancies, and autoimmune diseases. The pathogenesis of sHLH involves the excessive activation and proliferation of macrophages and cytotoxic T cells, leading to a cytokine storm. This cytokine storm is characterized by the overproduction of pro-inflammatory cytokines, including interferon-gamma (IFN-γ), TNF-α, and interleukins (IL-1, IL-6, IL-18), which contribute to widespread tissue damage and multiorgan dysfunction. sHLH presents with a spectrum of clinical manifestations that reflect the systemic inflammatory response^[14]^. Common symptoms include persistent high fever, cytopenias (anemia, thrombocytopenia, and leukopenia), hepatosplenomegaly, and lymphadenopathy. Laboratory findings often show hyperferritinemia, hypertriglyceridemia, and elevated levels of soluble interleukin-2 receptor (sCD25). Liver function abnormalities and coagulopathy are also frequent. The nonspecific and variable nature of these symptoms can complicate the diagnosis, necessitating a high index of suspicion, particularly in patients with underlying conditions like leukemia and HIV. The diagnosis of sHLH is challenging due to its overlap with other inflammatory and infectious conditions. The HLH-2004 diagnostic criteria, initially developed for primary HLH, are often used for diagnosing sHLH^[15]^. According to these criteria, a diagnosis can be made if at least five of the following eight criteria are met:

1. Fever
2. Splenomegaly
3. Cytopenias affecting at least two lineages in the peripheral blood
4. Hypertriglyceridemia and/or hypofibrinogenemia
5. Hemophagocytosis in bone marrow, spleen, or lymph nodes
6. Low or absent natural killer (NK) cell activity
7. Hyperferritinemia
8. Elevated soluble CD25 (sCD25) levels

Given the complexity of sHLH, it is crucial to consider the clinical context and underlying conditions when applying these criteria. sHLH can be triggered by a variety of factors, including infections (viral, bacterial, fungal, and parasitic), malignancies (particularly hematologic cancers such as leukemia and lymphoma), autoimmune diseases (such as systemic lupus erythematosus), and certain medications^[16]^. In the context of leukemia and HIV, the risk of sHLH is heightened due to the combined effects of immune dysregulation, chronic inflammation, and increased susceptibility to infections. The interplay between these factors can create a pro-inflammatory environment conducive to the development of sHLH. The management of sHLH involves addressing the underlying trigger, controlling the hyperinflammatory response, and supporting organ function^[17]^. Treatment regimens typically include immunosuppressive and cytotoxic therapies such as corticosteroids, etoposide, and cyclosporine. Intravenous immunoglobulin (IVIG) and biological agents targeting specific cytokines, such as anakinra (IL-1 receptor antagonist) and tocilizumab (IL-6 receptor antagonist), may also be used. In patients with leukemia, chemotherapy directed at the malignancy can help mitigate the inflammatory process. For HIV-positive patients, antiretroviral therapy (ART) is essential to control viral replication and improve immune function.

The prognosis of sHLH is variable and depends on the underlying cause, the timeliness of diagnosis, and the effectiveness of treatment^[18]^. Without prompt and appropriate management, sHLH can rapidly progress to multiorgan failure and death. However, with early recognition and targeted therapy, the outcomes can be significantly improved. Monitoring for treatment response and potential complications is crucial for optimizing patient outcomes. Managing sHLH in leukemia patients with HIV presents unique challenges^[19]^. The overlapping symptoms of sHLH, leukemia, and HIV-related complications can obscure the diagnosis. Additionally, the immunosuppressive treatments required for sHLH can exacerbate the immunocompromised state, increasing the risk of opportunistic infections. Drug interactions between chemotherapy, ART, and immunosuppressive agents further complicate treatment protocols. A multidisciplinary approach involving specialists in hematology, infectious diseases, and immunology is essential for effective management. Early recognition of sHLH is critical for improving patient outcomes^[20]^. Given the high mortality associated with delayed or missed diagnosis, healthcare providers must maintain a high level of vigilance for sHLH in high-risk populations, including leukemia patients with HIV. Regular monitoring of clinical and laboratory parameters, coupled with a proactive approach to managing potential triggers, can facilitate early intervention and reduce the risk of severe complications.

### Pathophysiology of HLH in HIV-positive leukemia patients

HLH is a life-threatening hyperinflammatory syndrome characterized by excessive immune activation and multi-organ dysfunction. In HIV-positive leukemia patients, the interplay between viral-driven immune dysregulation, malignant hematopoiesis, and metabolic stress creates a unique pathophysiological landscape that predisposes these individuals to HLH^[13]^.

### A storm of immune dysregulation

At the core of HLH is an uncontrolled immune response, where activated T-cells and macrophages fail to regulate their inflammatory output. In HIV-positive individuals, chronic viral replication results in persistent immune activation, despite an overall decline in CD4+ T-cell numbers. This paradoxical state – where the immune system is both hyperactive and impaired – creates fertile ground for HLH to emerge. Leukemia further compounds this immune dysfunction. Malignant hematopoietic cells can produce aberrant cytokines, disrupt normal immune regulation, and impair antigen presentation. As leukemia progresses, cytotoxic T-cells become increasingly dysfunctional, losing their ability to eliminate activated macrophages. This loss of immune control fuels a vicious cycle of inflammation, where macrophages engulf healthy hematopoietic cells in an unrestrained process known as hemophagocytosis^[14,15]^.

### The cytokine storm

One of the defining features of HLH is the overwhelming surge of proinflammatory cytokines, often referred to as a “cytokine storm.” In HIV-positive leukemia patients, this storm is fueled by a convergence of viral, malignant, and immune-driven signals. Interferon-gamma (IFN-γ) plays a central role in this process, acting as a powerful activator of macrophages. Once activated, macrophages release TNF-α, IL-6, and interleukin-1β (IL-1β), all of which contribute to fever, hepatosplenomegaly, and systemic inflammation. Additionally, soluble CD25 (sCD25), a marker of T-cell activation, becomes markedly elevated, signaling the loss of immune regulation. This uncontrolled inflammation is particularly devastating in leukemia patients, whose bone marrow is already compromised. The excessive cytokine release disrupts normal hematopoiesis, exacerbating cytopenias and increasing the risk of life-threatening infections^[16]^.

### The role of hematologic and metabolic dysfunctions

HLH in leukemia patients is often accompanied by profound hematologic abnormalities. The bone marrow, overwhelmed by malignant cells and activated macrophages, becomes a battlefield where normal blood cell production collapses. Red blood cells, platelets, and neutrophils are phagocytosed at an accelerated rate, leading to anemia, thrombocytopenia, and neutropenia. This rapid destruction of blood cells contributes to severe fatigue, bleeding tendencies, and heightened susceptibility to infections – hallmarks of HLH. Metabolic disturbances further complicate this landscape. HIV infection and leukemia both alter glucose metabolism, leading to insulin resistance and mitochondrial dysfunction. Adipose tissue-derived cytokines (adipokines) may also play a role, particularly in individuals with abnormal body mass index (BMI). Obesity, for example, is known to promote low-grade inflammation, which could amplify the cytokine storm of HLH. Conversely, severe malnutrition, often observed in advanced HIV cases, may weaken immune defenses, making patients more vulnerable to the unchecked spread of inflammatory damage^[17]^.

### Triggering the final cascade

In HIV-positive leukemia patients, HLH can be triggered by multiple factors, often in combination. Opportunistic infections such as cytomegalovirus (CMV), Epstein–Barr virus (EBV), and mycobacterium tuberculosis frequently act as catalysts, pushing an already fragile immune system into hyperactivation. These infections activate toll-like receptors (TLRs) on antigen-presenting cells, further amplifying cytokine production and hemophagocytosis. Leukemia itself can act as a direct trigger. Certain subtypes, particularly acute lymphoblastic leukemia (ALL) and acute myeloid leukemia (AML), are notorious for their association with HLH. Malignant blasts can secrete inflammatory mediators that mimic HLH-like responses, making differentiation between primary malignancy and HLH challenging^[18]^.

### The perfect storm

The overlapping features of HLH, HIV, and leukemia make diagnosis particularly difficult. Fever, cytopenias, and hepatosplenomegaly – hallmark signs of HLH – are also common in advanced HIV and leukemia. Biomarkers such as ferritin, soluble IL-2 receptor (sCD25), and CXCL9 (a chemokine induced by IFN-γ) can help differentiate HLH from other inflammatory conditions. However, the rapid progression of HLH necessitates early recognition and intervention. Treatment strategies must address all three underlying conditions simultaneously. ART remains the cornerstone of HIV management, while chemotherapy or targeted therapy is essential for leukemia control. HLH-specific therapies, including corticosteroids, etoposide, and cytokine inhibitors such as anakinra (IL-1 receptor antagonist) or tocilizumab (IL-6 receptor blocker), are critical for dampening the hyperinflammatory response^[19]^.

### Risk stratification

Risk stratification in HLH requires a multifactorial approach, integrating underlying disease burden, immune function, and inflammatory response. In HIV-positive leukemia patients, certain factors heighten susceptibility to HLH, necessitating early intervention:

#### Advanced HIV disease

Patients with low CD4+ T-cell counts (<200 cells/μL) and uncontrolled viral replication (high HIV RNA load) are at greater risk of immune dysregulation, making HLH more likely. Co-infections such as CMV, EBV, Mycobacterium tuberculosis, and fungal pathogens act as potent triggers, exacerbating cytokine dysregulation^[20]^.

#### Leukemia subtype and disease stage

Acute leukemias (AML, ALL) carry a higher risk due to aggressive disease progression and the production of inflammatory cytokines. Relapsed or refractory leukemia is associated with increased macrophage activation, further predisposing patients to HLH.

#### Chemotherapy-induced immune dysregulation

Cytotoxic agents suppress normal hematopoiesis, leading to profound neutropenia and increasing susceptibility to HLH. Immunomodulatory therapies (e.g. checkpoint inhibitors) may paradoxically trigger HLH by inducing excessive immune activation^[21]^.

#### Elevated inflammatory and hematologic markers

Extreme hyperferritinemia (>10 000 µg/L), rising D-dimer levels, and high lactate dehydrogenase correlate with severe HLH. Severe pancytopenia signals extensive bone marrow infiltration by activated macrophages^[22]^.

#### Metabolic and nutritional factors

Obesity-related inflammation (elevated adipokines) can worsen cytokine storm, while severe malnutrition can impair immune resilience, making HLH more devastating. Liver dysfunction (elevated transaminases, hyperbilirubinemia) and coagulopathy indicate systemic inflammatory spread^[23]^.

### Leukemia and HIV: A dual challenge

Leukemia is a group of cancers originating in the bone marrow and blood, characterized by the uncontrolled proliferation of abnormal white blood cells. These malignant cells disrupt normal hematopoiesis, leading to impaired production of healthy blood cells and subsequent immunosuppression. Leukemia patients often experience increased susceptibility to infections, anemia, bleeding tendencies, and other complications due to bone marrow failure. The immune dysregulation in leukemia is further exacerbated by the disease itself and the aggressive chemotherapies used for treatment, which can cause profound immunosuppression^[23–25]^. HIV specifically targets the immune system, attacking CD4+ T cells, macrophages, and dendritic cells. This progressive depletion of CD4+ T cells leads to a weakened immune system, making individuals more susceptible to opportunistic infections and certain malignancies. Without ART, HIV advances to acquired immunodeficiency syndrome, characterized by severe immunosuppression and life-threatening opportunistic infections and cancers. Even with ART, patients may still face chronic immune activation and residual immune dysfunction^[26–28]^. When leukemia and HIV coexist, the synergistic effects on the immune system are profound. Both conditions independently compromise immune function, and their coexistence can lead to compounded immunosuppression. Leukemia treatment often involves chemotherapies that further deplete immune cells, while HIV-induced immune suppression impairs the body’s ability to respond to infections and malignancies. This dual challenge significantly increases the risk of severe infections, opportunistic diseases, and inflammatory complications such as sHLH^[29,30]^. Studies by Patel *et al*^[31]^ reported an increased incidence of cancer among HIV-infected persons compared with the general population with a 2.5-fold increase in leukemia frequency. Furthermore, Kaner *et al*^[32]^ reported that leukemia (such as AML) in patients living with HIV presented at a younger age compared with HIV-negative patients (median age 56 vs. 96 years). Leukemia among other diseases, may become more prevalent in patients with HIV as this population ages^[33]^.

Leukemia patients with HIV face an extraordinarily high risk of infections due to their severely compromised immune systems. Common pathogens include bacterial, viral, fungal, and parasitic agents, many of which can cause opportunistic infections that are rare in immunocompetent individuals. These infections can trigger or exacerbate inflammatory responses, leading to conditions like sHLH. Recent studies by Tabaja *et al*^[34]^ have posited that at least one infectious agent in HIV-infected patients was thought to trigger HLH in 78% of the patients surveyed including Epstein–Barr virus (26%), human herpes virus (21%) and Histoplasma capsulatum (17%). Effective infection control and prophylactic measures are essential to manage this increased risk^[35,36]^. The treatment of leukemia in HIV-positive patients is complicated by the potential for drug interactions between chemotherapeutic agents and ART^[37]^. Chemotherapy can induce severe immunosuppression, increasing the risk of infections and complicating the management of HIV. Conversely, ART can have toxicities and side effects that overlap with those of chemotherapy, potentially leading to increased morbidity. Balancing the effective treatment of both leukemia and HIV requires careful consideration of drug interactions and close monitoring of patient response. Both leukemia and HIV can lead to significant inflammatory complications, including sHLH. HIV infection is associated with chronic immune activation and inflammation, even in the presence of ART^[38]^. Leukemia can also drive inflammatory responses due to the malignant proliferation of immune cells and the cytokine release associated with the disease and its treatment. When these inflammatory processes converge, the risk of developing hyperinflammatory syndromes like sHLH is heightened, necessitating vigilant monitoring and early intervention. Managing leukemia in HIV-positive patients requires a multidisciplinary approach involving hematologists, oncologists, infectious disease specialists, and immunologists^[39]^. This collaborative care model ensures comprehensive management of both conditions, addressing the complexities of dual immunosuppression and the heightened risk of complications. Personalized treatment plans that consider the unique interactions between leukemia and HIV therapies can optimize patient outcomes and reduce the risk of adverse effects.

Nutritional status plays a critical role in the immune function of leukemia patients with HIV^[40]^. Both malnutrition and obesity can impact immune responses, influencing the risk and severity of complications like sHLH. Malnutrition, common in advanced HIV and aggressive leukemia treatment, impairs immune function and increases susceptibility to infections. Conversely, obesity, which can be exacerbated by ART, promotes chronic inflammation and may contribute to the hyperinflammatory state seen in sHLH. Addressing nutritional status through tailored interventions is crucial for optimizing immune function and overall health. The dual diagnosis of leukemia and HIV can have profound psychological and social impacts on patients^[41]^. The stigma associated with HIV, combined with the fear and uncertainty of a leukemia diagnosis, can lead to significant emotional distress. Comprehensive care should include psychological support to address these challenges, along with social services to help patients navigate the complexities of their treatment regimens and access necessary resources. Ensuring patients have a strong support system can improve adherence to treatment and overall quality of life.

### BMI: An overlooked factor in sHLH risk

BMI is a widely used measure to assess an individual’s nutritional status, calculated as weight in kilograms divided by the square of height in meters (kg/m^2^). A BMI of less than 18.5 is classified as underweight, indicating potential malnutrition. In the context of leukemia patients living with HIV, low BMI is a significant concern due to the heightened metabolic demands and the impact of both the malignancy and the viral infection on the body’s nutritional reserves^[42]^. Malnutrition is prevalent among individuals with chronic illnesses, particularly those with conditions that impair nutrient absorption, increase metabolic requirements, or reduce appetite. Malnutrition profoundly impacts immune function, compromising both innate and adaptive immune responses. Protein-energy malnutrition leads to reduced production and function of immune cells, including T lymphocytes, B lymphocytes, and macrophages. This weakened immune response heightens susceptibility to infections, impairs wound healing, and reduces the efficacy of vaccines. In leukemia patients with HIV, an already compromised immune system due to HIV-induced CD4+ T cell depletion is further weakened by malnutrition, exacerbating the risk of opportunistic infections and inflammatory complications such as sHLH^[43]^. Malnutrition affects the body’s ability to regulate inflammatory responses. Nutrient deficiencies impair the production of anti-inflammatory cytokines and enhance the secretion of pro-inflammatory cytokines like TNF-α, IL-1, and IL-6. This imbalance can lead to chronic low-grade inflammation, which, in the context of sHLH, can exacerbate the cytokine storm. In leukemia patients with HIV, malnutrition-induced inflammatory dysregulation can trigger or worsen sHLH, leading to severe clinical outcomes. Malnutrition also directly affects hematopoiesis, leading to anemia, leukopenia, and thrombocytopenia^[44]^. These hematologic abnormalities are particularly detrimental in leukemia patients, who already suffer from impaired blood cell production due to the malignancy and its treatment. Anemia and leukopenia can reduce the body’s ability to fight infections and respond to stress, while thrombocytopenia increases the risk of bleeding complications. In HIV-positive individuals, where the bone marrow may already be compromised by viral infection and ART toxicity, the added burden of malnutrition can severely impact patient outcomes.

The clinical signs of malnutrition in leukemia patients with HIV include weight loss, muscle wasting, weakness, and fatigue. These patients may also present with micronutrient deficiencies, leading to symptoms such as glossitis, cheilosis, dermatitis, and impaired wound healing. The combination of chronic disease, increased metabolic demands, and reduced nutritional intake creates a vicious cycle, further exacerbating malnutrition and its associated complications^[45–49]^. Accurate assessment of nutritional status is essential for identifying malnutrition in leukemia patients with HIV^[50]^. This involves comprehensive evaluations, including anthropometric measurements (BMI, weight changes, mid-upper arm circumference), biochemical markers (serum albumin, prealbumin, transferrin), and clinical assessments (physical examination, dietary history). Identifying and addressing the underlying causes of malnutrition, such as gastrointestinal issues, infections, or chemotherapy-related side effects, is crucial for effective intervention. Addressing malnutrition in leukemia patients with HIV requires a multidisciplinary approach^[51]^. Nutritional interventions should be tailored to meet the individual’s specific needs, focusing on increasing caloric and protein intake, correcting micronutrient deficiencies, and managing symptoms that affect appetite and absorption. Oral nutritional supplements, enteral nutrition, or parenteral nutrition may be necessary in severe cases. Additionally, managing gastrointestinal symptoms, providing appetite stimulants, and ensuring adequate hydration are essential components of nutritional support.

Optimizing nutritional status is crucial in managing sHLH in leukemia patients with HIV^[51]^. Adequate nutrition supports immune function, enhances the body’s ability to respond to infections and inflammation, and improves tolerance to treatments. Nutritional support can help mitigate the impact of the cytokine storm characteristic of sHLH, potentially reducing its severity and improving patient outcomes. Early intervention and ongoing monitoring of nutritional status are key strategies in the comprehensive management of these patients. Effective management of malnutrition in leukemia patients with HIV requires a collaborative effort among healthcare providers, including oncologists, infectious disease specialists, dietitians, and social workers^[52]^. This multidisciplinary team approach ensures that all aspects of the patient’s nutritional needs are addressed, from dietary counseling and supplementation to managing treatment-related side effects and providing psychosocial support. Regular follow-up and adjustment of nutritional interventions based on the patient’s progress are essential for sustained improvement in nutritional status and overall health.

### High BMI and obesity

High BMI, defined as a BMI of 25–29.9 kg/m^2^ for overweight and 30 kg/m^2^ or higher for obesity, is a growing health concern worldwide. In the context of leukemia patients living with HIV, high BMI can complicate the clinical management and prognosis of these individuals^[53]^. The prevalence of obesity has been increasing globally, driven by factors such as sedentary lifestyles, high-calorie diets, and genetic predispositions. Among individuals with chronic conditions like leukemia and HIV, the metabolic changes induced by disease and treatment can further contribute to weight gain and obesity^[54]^. Obesity is associated with chronic low-grade inflammation, which can alter immune function^[55]^. Adipose tissue, particularly visceral fat, acts as an endocrine organ, secreting pro-inflammatory cytokines such as TNF-α, IL-6, and leptin. These cytokines promote a state of systemic inflammation, impairing both innate and adaptive immune responses. In leukemia patients with HIV, this chronic inflammatory state can exacerbate immune dysregulation and increase susceptibility to infections and inflammatory complications such as sHLH. Obesity leads to metabolic dysregulation, characterized by insulin resistance, hyperglycemia, and dyslipidemia. These metabolic disturbances can have significant implications for leukemia patients with HIV^[56]^. Insulin resistance and hyperglycemia can impair immune function, reduce the efficacy of chemotherapy, and increase the risk of infections. Dyslipidemia, characterized by elevated levels of triglycerides and low-density lipoprotein (LDL) cholesterol, is also associated with increased inflammatory responses. These metabolic abnormalities contribute to the overall disease burden and complicate the management of both leukemia and HIV.

The chronic inflammation associated with obesity plays a critical role in the pathogenesis of sHLH. Adipose tissue-derived cytokines enhance the production of pro-inflammatory mediators, contributing to the cytokine storm characteristic of sHLH^[57]^. This hyperinflammatory state can lead to widespread activation of macrophages and T lymphocytes, resulting in hemophagocytosis and multiorgan damage. In leukemia patients with HIV, where the immune system is already compromised, obesity-induced inflammation can significantly increase the risk and severity of sHLH. Leukemia patients with HIV who are obese often present with a range of clinical complications. These can include increased fatigue, reduced mobility, and higher incidence of comorbid conditions such as cardiovascular disease, diabetes, and hypertension^[58]^. Obesity can also complicate the physical examination and diagnostic imaging, making it more challenging to detect disease progression and complications. The additional weight can place extra strain on the cardiovascular system, exacerbating the risk of thromboembolic events and other cardiovascular complications. Assessing and managing obesity in leukemia patients with HIV presents several challenges^[55]^. Standard diagnostic tools such as BMI may not fully capture the extent of metabolic and inflammatory abnormalities associated with obesity. Body composition analysis, including measurements of visceral fat and muscle mass, can provide a more accurate assessment of obesity-related risks. Additionally, the overlapping symptoms of obesity-related comorbidities and complications of leukemia and HIV can complicate the diagnostic process, requiring careful and comprehensive evaluation. Addressing obesity in leukemia patients with HIV requires a multifaceted approach. Nutritional interventions should focus on reducing caloric intake, improving dietary quality, and promoting weight loss through balanced, nutrient-dense diets. Physical activity, tailored to the patient’s abilities and health status, can help reduce body fat, improve metabolic health, and enhance overall well-being. Behavioral interventions, including counseling and support groups, can help patients adopt and maintain healthy lifestyle changes. Managing obesity effectively can reduce the inflammatory burden and improve immune function, potentially mitigating the risk of sHLH. In some cases, pharmacological or surgical interventions may be necessary to manage obesity. Medications such as metformin, GLP-1 receptor agonists, and SGLT2 inhibitors can aid in weight loss and improve metabolic parameters. Bariatric surgery, including procedures like gastric bypass and sleeve gastrectomy, can be considered for severely obese patients who do not respond to conservative measures. These interventions can result in significant and sustained weight loss, improving metabolic health and reducing the risk of obesity-related complications. However, the potential benefits must be weighed against the risks, particularly in immunocompromised patients with complex health conditions.

### Mechanisms linking BMI and sHLH

BMI, whether low or high, plays a significant role in modulating immune responses and inflammatory processes, thereby influencing the risk and progression of sHLH.

### Low BMI and malnutrition

Malnutrition, often indicated by a low BMI, profoundly affects immune function. Protein-energy malnutrition leads to atrophy of lymphoid tissues, decreased production of immunoglobulins, and impaired phagocytic activity of macrophages^[59]^. The resulting immunodeficiency reduces the body’s ability to control infections, which can trigger or exacerbate sHLH. In malnourished individuals, the immune system’s capacity to regulate inflammatory responses is compromised, making them more susceptible to the hyperinflammatory state characteristic of sHLH. Malnutrition alters the balance of pro-inflammatory and anti-inflammatory cytokines. Deficiencies in essential nutrients, such as vitamins A, C, D, and E, and minerals like zinc and selenium, impair the synthesis and function of anti-inflammatory cytokines while promoting the production of pro-inflammatory cytokines. This imbalance can contribute to the cytokine storm seen in sHLH, where excessive levels of TNF-α, IL-6, and IFN-γ drive systemic inflammation and hemophagocytosis. Leukemia and HIV further complicate the scenario by causing bone marrow suppression, which is exacerbated by malnutrition^[60]^. Reduced production of hematopoietic cells leads to cytopenias, a hallmark of sHLH. The combination of nutritional deficiencies and bone marrow dysfunction impairs the body’s ability to produce and maintain adequate levels of immune cells, increasing the risk of sHLH in malnourished patients.

### High BMI and obesity

Obesity, indicated by a high BMI, is associated with chronic low-grade inflammation. Adipose tissue, especially visceral fat, secretes a range of pro-inflammatory cytokines and adipokines, including TNF-α, IL-6, and leptin. This chronic inflammatory state can prime the immune system for an exaggerated response to infections or other triggers, increasing the likelihood of developing sHLH. The persistent inflammatory milieu in obese individuals can amplify the cytokine storm and tissue damage associated with sHLH. Obesity leads to metabolic dysregulation; characterized by insulin resistance, hyperglycemia, and dyslipidemia^[61]^. These metabolic abnormalities can further impact immune function and inflammatory responses. Insulin resistance and hyperglycemia impair the functionality of immune cells, reducing their ability to combat infections effectively. Dyslipidemia, with elevated levels of triglycerides and LDL cholesterol, is also linked to increased production of pro-inflammatory cytokines, contributing to the hyperinflammatory state of sHLH^[62]^. Adipokines, such as leptin and adiponectin, play significant roles in immune modulation. Leptin, which is elevated in obesity, promotes pro-inflammatory immune responses, enhancing the production of Th1 cytokines and reducing regulatory T-cell activity. This can lead to an increased risk of hyperinflammatory conditions like sHLH. Conversely, adiponectin, which has anti-inflammatory properties, is often reduced in obesity, further skewing the balance towards a pro-inflammatory state.

### Combined effects in leukemia and HIV patients

In leukemia patients with HIV, the interplay between BMI, immune function, and sHLH is particularly complex. Both low and high BMI can exacerbate the immune dysregulation caused by leukemia and HIV. Malnutrition impairs immune cell production and function, while obesity drives chronic inflammation and metabolic disturbances. These factors, combined with the underlying immunosuppression from HIV and the hematologic impact of leukemia, create a perfect storm for the development of sHLH. For malnourished patients, nutritional support and supplementation are critical to restoring immune function and reducing the risk of sHLH. In obese patients, interventions aimed at reducing adipose tissue inflammation and improving metabolic health, such as weight loss, dietary modifications, and potentially pharmacotherapy, can help mitigate the inflammatory and metabolic drivers of sHLH. Personalized treatment plans that address the specific nutritional and metabolic needs of leukemia patients with HIV are essential for preventing and managing sHLH^[63]^.

### Clinical implications and management

Both low and high BMI contribute to immune dysregulation, increasing the risk of sHLH in leukemia patients living with HIV^[64]^. Malnutrition leads to immunosuppression and impaired immune responses, while obesity induces chronic inflammation and metabolic disturbances. These BMI-related factors exacerbate the underlying immune dysfunction caused by leukemia and HIV, creating a heightened susceptibility to sHLH. Recognizing these risks is crucial for early intervention and management. The coexistence of malnutrition or obesity with leukemia and HIV complicates disease management. Malnourished patients may have poor tolerance to chemotherapy and ART, leading to suboptimal treatment outcomes. Obese patients, on the other hand, may experience difficulties with drug dosing, increased side effects, and a higher incidence of comorbidities such as diabetes and cardiovascular disease. These factors necessitate personalized treatment approaches that consider the patient’s nutritional and metabolic status. BMI-related immune dysregulation can mask or mimic the symptoms of leukemia and HIV, complicating the diagnostic process. Symptoms of sHLH, such as fever, cytopenias, and organomegaly, overlap with those of leukemia and HIV-related infections. Additionally, chronic inflammation in obese patients can obscure the clinical picture, making it challenging to distinguish between disease progression and inflammatory complications. Comprehensive diagnostic evaluations, including advanced imaging, laboratory tests, and immune profiling, are essential for accurate diagnosis^[65,66]^.

## Management strategies

### Nutritional support for malnourished patients

Effective management of malnutrition involves a multidisciplinary approach to restore nutritional status and support immune function. Key strategies include:

- Nutritional assessment: Regular monitoring of weight, BMI, and biochemical markers to identify and address malnutrition early.
- Dietary interventions: High-calorie, high-protein diets to meet the increased metabolic demands of leukemia and HIV. Oral nutritional supplements or enteral nutrition may be necessary for patients with severe malnutrition.
- Micronutrient supplementation: Providing vitamins and minerals, such as vitamins A, C, D, E, zinc, and selenium, to correct deficiencies and support immune function.
- Management of gastrointestinal symptoms: Treating nausea, vomiting, and diarrhea, which can impair nutrient absorption and intake^[28]^.

### Weight management for obese patients

Addressing obesity involves interventions to reduce inflammation and improve metabolic health, thereby mitigating the risk of sHLH. Key strategies include:

- Nutritional counseling: Developing individualized, calorie-controlled meal plans that emphasize nutrient-dense foods and limit high-fat, high-sugar foods.
- Physical activity: Encouraging regular exercise tailored to the patient’s abilities, which can help reduce body fat, improve cardiovascular health, and enhance overall well-being.
- Pharmacotherapy: Considering medications such as metformin, GLP-1 receptor agonists, or SGLT2 inhibitors to assist with weight loss and metabolic control.
- Bariatric surgery: Evaluating the suitability of bariatric surgery for severely obese patients who do not respond to conservative measures, weighing the potential benefits against the risks^[29]^.

### Managing sHLH

Timely recognition and treatment of sHLH are critical to improving outcomes. Management strategies include:

- Immunomodulatory therapy: Using corticosteroids, IVIG, or biologics (e.g. anakinra, tocilizumab) to suppress the hyperinflammatory response and control cytokine levels.
- Infection control: Aggressive treatment of underlying infections with appropriate antibiotics, antivirals, or antifungals, given the high risk of infections triggering sHLH.
- Supportive care: Providing blood transfusions, antipyretics, and other supportive measures to manage symptoms and complications of sHLH.
- Close monitoring: Regular monitoring of clinical and laboratory parameters to assess response to treatment and detect any signs of disease progression or relapse^[30]^.

### Multidisciplinary care

Effective management of leukemia patients with HIV and BMI-related complications requires a coordinated, multidisciplinary approach. Key components include:

- Oncologists: Overseeing cancer treatment, managing chemotherapy-related side effects, and coordinating care with other specialists.
- Infectious disease specialists: Managing HIV and related infections, adjusting ART regimens as needed, and providing prophylactic measures to prevent opportunistic infections.
- Dietitians: Developing and monitoring nutritional interventions, providing dietary counseling, and addressing specific nutritional needs.
- Endocrinologists: Managing metabolic complications, such as diabetes and dyslipidemia, particularly in obese patients.
- Physical therapists: Designing and supervising exercise programs to improve physical function and support weight management.
- Social workers and psychologists: Providing psychosocial support, addressing mental health issues, and assisting with access to resources and services^[31]^.

### Future directions

Future research should focus on elucidating the precise mechanisms linking BMI to immune dysregulation and sHLH in leukemia patients with HIV. Investigating how specific BMI-related factors, such as adipokines in obesity or micronutrient deficiencies in malnutrition, influence immune responses can provide insights into targeted therapeutic approaches. Understanding the interplay between BMI, cytokine profiles, and immune cell function will be crucial for developing novel interventions. Advancements in personalized medicine could revolutionize the management of BMI-related complications in leukemia patients with HIV. By integrating genetic, metabolic, and immunological data, personalized treatment plans can be developed to address the unique needs of each patient. Precision nutrition and pharmacogenomics could play significant roles in tailoring dietary and pharmacological interventions to optimize outcomes and minimize adverse effects^[32]^.

### Innovative therapeutic approaches

Developing targeted therapies that specifically address the inflammatory and metabolic pathways influenced by BMI could offer new treatment options for managing sHLH. For instance, novel drugs that selectively inhibit pro-inflammatory cytokines or modulate adipokine activity could help manage chronic inflammation and reduce the risk of sHLH. Research into the role of immune checkpoint inhibitors and other emerging therapies could also provide new avenues for treatment. Future research should explore advanced nutritional interventions to better manage malnutrition and obesity in this population. This includes investigating the efficacy of personalized dietary plans, functional foods, and nutraceuticals in improving immune function and reducing inflammation. Additionally, evaluating the impact of specific micronutrient supplementation on immune health and sHLH risk could lead to more effective nutritional strategies. New approaches to metabolic modulation, including pharmacological agents that target insulin resistance and dyslipidemia, could improve the management of obesity-related inflammation and sHLH. Investigating the impact of weight loss interventions, such as bariatric surgery, on immune function and sHLH outcomes in this population may also provide valuable insights^[33]^.

### Enhancing diagnostic and monitoring tools

Identifying and validating biomarkers associated with BMI-related immune dysregulation and sHLH could enhance early diagnosis and treatment. Research into novel biomarkers for detecting sHLH, assessing immune function, and monitoring the impact of BMI-related factors on disease progression will be critical. Advances in diagnostic imaging and laboratory techniques could also improve the accuracy and timeliness of diagnosis. The use of wearable technologies and remote monitoring tools could provide real-time data on patient health, including weight, activity levels, and physiological parameters. Integrating these technologies into clinical practice could facilitate early detection of complications, personalized monitoring, and timely adjustments to treatment plans. This approach could improve patient outcomes and quality of life by allowing for more proactive and responsive care^[30]^.

### Improving multidisciplinary care models

Developing integrated care pathways that encompass all aspects of managing leukemia, HIV, and BMI-related complications will be essential. In recent studies by Abdelhay *et al*^[65]^ costs of HLH approached >$100 000 USD with a ~30-day maximum length of hospital stay per patient, where infection and malignancies infection comprised 21%–28% of reported mortality rates, respectively, the over 16 000 HLH admissions surveyed. Thus, these pathways should involve collaboration among oncologists, infectious disease specialists, dietitians, endocrinologists, and other healthcare professionals to ensure comprehensive and coordinated care. Standardizing care protocols and guidelines can improve the consistency and quality of treatment across different healthcare settings. Future research should also focus on patient-centered approaches that address the psychosocial and behavioral aspects of managing BMI-related complications. Incorporating patient feedback into care models can enhance the effectiveness of treatment and support overall well-being^[31]^.

### Policy and access to care

Advocating for policies that improve access to care, including nutritional support, weight management programs, and comprehensive cancer care, is crucial. Ensuring that patients have access to necessary resources and interventions can significantly impact their health outcomes. Policy initiatives should also focus on reducing healthcare disparities and improving equity in care for underserved populations. Enhancing education and training for healthcare providers on the management of BMI-related complications in leukemia and HIV patients is essential. Continuing medical education programs should include updates on the latest research, best practices, and emerging therapies. Training programs should also emphasize the importance of a multidisciplinary approach and effective communication among healthcare team members^[32,33]^.

## Conclusion

The interplay between BMI and sHLH in leukemia patients living with HIV presents a multifaceted challenge that significantly impacts patient outcomes. Both low and high BMI contribute to immune dysregulation, which is a critical factor in the development and progression of sHLH. Malnutrition, often indicated by a low BMI, impairs immune function and exacerbates the risk of infections, while obesity, marked by a high BMI, drives chronic inflammation and metabolic disturbances that can precipitate sHLH. Managing these complications requires a comprehensive and nuanced approach. For malnourished patients, targeted nutritional support, including dietary interventions and micronutrient supplementation, is essential for restoring immune function and preventing sHLH. For obese patients, strategies to reduce chronic inflammation and address metabolic abnormalities, such as weight management, dietary changes, and pharmacotherapy, are crucial for mitigating the risk of sHLH. Effective management also involves a multidisciplinary approach, integrating the expertise of oncologists, infectious disease specialists, dietitians, endocrinologists, and other healthcare professionals. This collaborative care model ensures that all aspects of the patient’s health, from disease treatment to nutritional and metabolic management, are addressed comprehensively.

## Data Availability

Not applicable as this is a review.

## Research registration unique identifying number (UIN)

Not applicable as this is a review.

## References

[1] Sepulveda FE de Saint Basile G. Hemophagocytic syndrome: primary forms and predisposing conditions. Curr Opin Immunol 2017;49:20–26. 28866302 10.1016/j.coi.2017.08.004

[2] Imashuku S Morimoto A Ishii E. Virus‐triggered secondary hemophagocytic lymphohistiocytosis. Acta Paediatr 2021;110:2729–36. 34096649 10.1111/apa.15973

[3] Obeagu EI Obeagu GU Ukibe NR. Anemia, iron, and HIV: decoding the interconnected pathways: a review. Medicine (Baltimore) 2024;103:e36937. 38215133 10.1097/MD.0000000000036937 PMC10783375

[4] Obeagu EI. Diagnostic and prognostic significance of mast cell markers in HIV/AIDS: current insights and future directions. Medicine (Baltimore) 2024;103:e38117. 38758896 10.1097/MD.0000000000038117 PMC11098248

[5] Christinelli HC Westphal G Junior NN. Nutritional status and body composition in individuals with overweight or obesity using usual and unusual indicators. Res Soc Dev 2021;10:e4910111339.

[6] Kiran S Kumar V Kumar S. Adipocyte, immune cells, and miRNA crosstalk: a novel regulator of metabolic dysfunction and obesity. Cells 2021;10:1004. 33923175 10.3390/cells10051004 PMC8147115

[7] Yu X PU H Voss M. Overview of anti-inflammatory diets and their promising effects on non-communicable diseases. Br J Nutr 2024;132:898–918. 10.1017/S0007114524001405 PMC11576095 39411832

[8] Shimizu M. Clinical features of cytokine storm syndrome. Cytokine Storm Syndrome 2024;1448:33–42. 10.1007/978-3-031-59815-9_4 39117806

[9] Muscogiuri G Pugliese G Laudisio D. The impact of obesity on immune response to infection: plausible mechanisms and outcomes. Obesity Rev 2021;22:e13216. 10.1111/obr.13216 33719175

[10] Karakike E Giamarellos-Bourboulis EJ. Macrophage activation-like syndrome: a distinct entity leading to early death in sepsis. Front Immunol 2019;10:55. 30766533 10.3389/fimmu.2019.00055 PMC6365431

[11] Bauchmuller K Manson JJ Tattersall R. Haemophagocytic lymphohistiocytosis in adult critical care. J Intensive Care Soc 2020;21:256–68. 32782466 10.1177/1751143719893865 PMC7401439

[12] Griffin G Shenoi S Hughes GC. Hemophagocytic lymphohistiocytosis: an update on pathogenesis, diagnosis, and therapy. Best Pract Res Clin Rheumatol 2020;34:101515. 32387063 10.1016/j.berh.2020.101515

[13] Castillo L Carcillo J. Secondary hemophagocytic lymphohistiocytosis and severe sepsis/systemic inflammatory response syndrome/multiorgan dysfunction syndrome/macrophage activation syndrome share common intermediate phenotypes on a spectrum of inflammation. Pediatr Crit Care Med 2009;10:387–92. 19325510 10.1097/PCC.0b013e3181a1ae08

[14] Hutchinson M Tattersall RS Manson JJ. Haemophagocytic lymphohisticytosis—an underrecognized hyperinflammatory syndrome. Rheumatology 2019;58:vi23–30. 31769857 10.1093/rheumatology/kez379 PMC6878843

[15] Sandler RD Carter S Kaur H. Haemophagocytic lymphohistiocytosis (HLH) following allogeneic haematopoietic stem cell transplantation (HSCT)—time to reappraise with modern diagnostic and treatment strategies? Bone Marrow Transplant 2020;55:307–16. 31455895 10.1038/s41409-019-0637-7 PMC6995779

[16] Filippone EJ Farber JL. Hemophagocytic lymphohistiocytosis: an update for nephrologists. Int Urol Nephrol 2016;48:1291–304. 27098410 10.1007/s11255-016-1294-z

[17] Carter SJ Tattersall RS Ramanan AV. Macrophage activation syndrome in adults: recent advances in pathophysiology, diagnosis and treatment. Rheumatology 2019;58:5–17. 29481673 10.1093/rheumatology/key006

[18] Chen J Wang X He P. Viral etiology, clinical and laboratory features of adult hemophagocytic lymphohistiocytosis. J Med Virol 2016;88:541–49. 26287378 10.1002/jmv.24359 PMC7166822

[19] Lee JC Logan AC. Diagnosis and management of adult malignancy-associated hemophagocytic lymphohistiocytosis. Cancers (Basel) 2023;15:1839. 36980725 10.3390/cancers15061839 PMC10046521

[20] Machado T Lawrence J Eriksson D. A need for greater awareness: a mixed methods study of patient and healthcare professional perspectives on the diagnosis journey for haemophagocytic lymphohistiocytosis (HLH). Hematology 2024;29:2358261. 38934707 10.1080/16078454.2024.2358261

[21] Obeagu EI. The relationship between body mass index and cytogenetic abnormalities in leukemia patients with HIV: a review. Elite J Haematol 2024;2:1–5.

[22] Obeagu EI Omar DM Omar U. Leukaemia burden in Africa. Int J Curr Res Biol Med 2023;1:17–22.

[23] Obeagu EI Gnanavel K. An insight on acute myeloid leukemia. Pediatr Perspect 2022;10:1-8.

[24] Obeagu EI Obeagu GU. The impact of obesity on overall survival in leukemia patients living with HIV: a review. Elite J Lab Med 2024;2:26–45.

[25] Obeagu EI. Obesity and treatment-related neurotoxicity in leukemia patients with advanced HIV. AIDS: a review. Elite J Med Sci 2024;2:31–39.

[26] Obeagu EI Obeagu GU. The nexus between obesity and leukemia progression in HIV-positive individuals: a review. Elite J Haematol 2024;2:180–98.

[27] Obeagu EI Elamin EA Obeagu GU. The impact of BMI on treatment outcomes in leukemia patients with HIV: a review. Elite J Haematol 2024;2:23–35.

[28] Obeagu EI. Body mass index and risk of leukemic transformation in HIV-positive patients with chronic lymphocytic leukemia: a review. Elite J Med 2024;2:22–31.

[29] Obeagu EI. Exploring the impact of body mass index on quality of life in leukemia patients living with HIV: a review. Elite J Haematol 2024;2:39–54.

[30] Obeagu EI. Body mass index changes during remission and relapse in leukemia patients living with HIV: a review. Elite J Lab Med 2024;2:32–40.

[31] Patel P Hanson DL Sullivan PS. Adult and adolescent spectrum of disease project and HIV outpatient study investigators. Incidence of types of cancer among HIV-infected persons compared with the general population in the United States, 1992-2003. Ann Intern Med 2008;148:728–36. 18490686 10.7326/0003-4819-148-10-200805200-00005

[32] Kaner J Thibaud S Sridharan A. HIV is associated with a high rate of unexplained multilineage cytopenias and portends a poor prognosis in myelodysplastic syndrome (MDS) and acute myeloid leukemia (AML). Blood 2016;128:4345.

[33] Aboulafia DM Meneses M Ginsberg S. Acute myeloid leukemia in patients infected with HIV-1. AIDS 2002;16:865–76. 11919488 10.1097/00002030-200204120-00006

[34] Tabaja H Kanj A El Zein S. A review of hemophagocytic lymphohistiocytosis in patients with HIV. Open Forum Infect Dis 2022;9:ofac071. 35308483 10.1093/ofid/ofac071 PMC8926004

[35] Obeagu EI. Understanding body mass index variations and clinical outcomes in leukemia patients with HIV. AIDS: a review. Elite J Health Sci 2024;2:59–72.

[36] Obeagu EI Obeagu GU. The impact of body mass index (BMI) on immune function in leukemia patients living with HIV: a review. Elite J Immunol 2024;2:73–92.

[37] Berretta M Caraglia M Martellotta F. Drug–drug interactions based on pharmacogenetic profile between highly active antiretroviral therapy and antiblastic chemotherapy in cancer patients with HIV infection. Front Pharmacol 2016;7:71. 27065862 10.3389/fphar.2016.00071 PMC4811911

[38] Lembas A Załęski A Peller M. Human immunodeficiency virus as a risk factor for cardiovascular disease. Cardiovasc Toxicol 2024;24:1–4. 37982976 10.1007/s12012-023-09815-4 PMC10838226

[39] Forghieri F Nasillo V Bettelli F. Acute myeloid leukemia in patients living with HIV infection: several questions, fewer answers. Int J Mol Sci 2020;21:1081. 32041199 10.3390/ijms21031081 PMC7036847

[40] Wu D Lewis ED Pae M. Nutritional modulation of immune function: analysis of evidence, mechanisms, and clinical relevance. Front Immunol 2019;9:3160. 30697214 10.3389/fimmu.2018.03160 PMC6340979

[41] Rodriguez-Penney AT Iudicello JE Riggs PK. HIV Neurobehavioral Research Program (HNRP) Group SP. Co-morbidities in persons infected with HIV: increased burden with older age and negative effects on health-related quality of life. AIDS Patient Care STDS 2013;27:5–16. 23305257 10.1089/apc.2012.0329 PMC3545369

[42] Vishnu P Aboulafia DM. Haematological manifestations of human immune deficiency virus infection. Br J Haematol 2015;171:695–709. 26452169 10.1111/bjh.13783

[43] Slots J. Oral viral infections of adults. Periodontol 2000 2009;49:60–86. 19152526 10.1111/j.1600-0757.2008.00279.x

[44] Bedha A Shindano T Hermans MP. Association between severe acute malnutrition in childhood and hematological disorders in adulthood: the Lwiro follow-up study in the Eastern Democratic Republic of the Congo. BMC Nutr 2023;9:128. 37951995 10.1186/s40795-023-00783-0 PMC10638766

[45] Anyiam AF Arinze-Anyiam OC Irondi EA. Distribution of ABO and rhesus blood grouping with HIV infection among blood donors in Ekiti State Nigeria. Medicine (Baltimore) 2023;102:e36342. 38013335 10.1097/MD.0000000000036342 PMC10681551

[46] Aizaz M Abbas FA Abbas A. Alarming rise in HIV cases in Pakistan: challenges and future recommendations at hand. Health Sci Rep 2023;6:e1450. 37520460 10.1002/hsr2.1450 PMC10375546

[47] Opeyemi AA Obeagu EI. Regulations of malaria in children with human immunodeficiency virus infection: a review. Medicine (Baltimore) 2023;102:e36166. 37986340 10.1097/MD.0000000000036166 PMC10659731

[48] Obeagu EI Obeagu GU Ede MO. Translation of HIV/AIDS knowledge into behavior change among secondary school adolescents in Uganda: a review. Medicine (Baltimore) 2023;102:e36599. 38065920 10.1097/MD.0000000000036599 PMC10713174

[49] Obeagu EI Obeagu GU. Platelet index ratios in HIV: emerging biomarkers for immune health and disease management. Medicine (Baltimore) 2024;103:e37576. 38518025 10.1097/MD.0000000000037576 PMC10956946

[50] Viani K Trehan A Manzoli B. Assessment of nutritional status in children with cancer: a narrative review. Pediatr Blood Cancer 2020;67:e28211. 32096326 10.1002/pbc.28211

[51] van Lieshout R Tick LW de Beer F. Medical nutrition therapy during intensive remission-induction treatment and hematopoietic stem cell transplantation in acute myeloid leukemia patients: hematologists’ experiences and perspectives. Clin Nutr ESPEN 2023;57:399–409. 37739686 10.1016/j.clnesp.2023.07.015

[52] Soto-perez-de-celis E Martínez-Peromingo J Chávarri-Guerra Y. Implementation of geriatric haematology programmes for the treatment of older people with haematological malignancies in low-resource settings. Lancet Healthy Longev 2021;2:e754–763. 36098032 10.1016/S2666-7568(21)00182-3

[53] Lange BJ Gerbing RB Feusner J. Mortality in overweight and underweight children with acute myeloid leukemia. Jama 2005;293:203–11. 15644547 10.1001/jama.293.2.203

[54] Annaloro C Airaghi L Saporiti G. Metabolic syndrome in patients with hematological diseases. Expert Rev Hematol 2012;5:439–58. 22992237 10.1586/ehm.12.35

[55] Khanna D Khanna S Khanna P. Obesity: a chronic low-grade inflammation and its markers. Cureus 2022;14:e22711. 35386146 10.7759/cureus.22711 PMC8967417

[56] da Cunha J Maselli LM Stern AC. Impact of antiretroviral therapy on lipid metabolism of human immunodeficiency virus-infected patients: old and new drugs. World J Virol 2015;4:56. 25964872 10.5501/wjv.v4.i2.56 PMC4419122

[57] Marques-Rocha JL Samblas M Milagro FI. Noncoding RNAs, cytokines, and inflammation-related diseases. FASEB J 2015;29:3595–611. 26065857 10.1096/fj.14-260323

[58] Dandachi D Geiger G Montgomery MW. Characteristics, comorbidities, and outcomes in a multicenter registry of patients with human immunodeficiency virus and coronavirus disease 2019. Clin Infect Dis 2021;73:e1964–72. 32905581 10.1093/cid/ciaa1339 PMC7499544

[59] Morales F Montserrat-de la Paz S Leon MJ. Effects of malnutrition on the immune system and infection and the role of nutritional strategies regarding improvements in children’s health status: a literature review. Nutrients 2023;16:1. 38201831 10.3390/nu16010001 PMC10780435

[60] Chinen J Shearer WT. Secondary immunodeficiencies, including HIV infection. J Allergy Clin Immunol 2010;125:S195–203. 20042227 10.1016/j.jaci.2009.08.040 PMC6151868

[61] Jung UJ Choi MS. Obesity and its metabolic complications: the role of adipokines and the relationship between obesity, inflammation, insulin resistance, dyslipidemia and nonalcoholic fatty liver disease. Int J Mol Sci 2014;15:6184–223. 24733068 10.3390/ijms15046184 PMC4013623

[62] Ahmed W Orasanu G Nehra V. High-density lipoprotein hydrolysis by endothelial lipase activates PPARα: a candidate mechanism for high-density lipoprotein-mediated repression of leukocyte adhesion. Circ Res 2006;98:490–98. 16439686 10.1161/01.RES.0000205846.46812.be PMC4231787

[63] Nobs SP Zmora N Elinav E. Nutrition regulates innate immunity in health and disease. Annu Rev Nutr 2020;40:189–219. 32520640 10.1146/annurev-nutr-120919-094440

[64] Rocco JM Laidlaw E Galindo F. Severe mycobacterial immune reconstitution inflammatory syndrome (IRIS) in advanced human immunodeficiency virus (HIV) has features of hemophagocytic lymphohistiocytosis and requires prolonged immune suppression. Clin Infect Dis 2023;76:e561–570. 36048425 10.1093/cid/ciac717 PMC10169423

[65] Abdelhay A Mahmoud AA Al Ali O. Epidemiology, characteristics, and outcomes of adult haemophagocytic lymphohistiocytosis in the USA, 2006-19: a national, retrospective cohort study. EClinicalMedicine 2023;62:102143. 37599909 10.1016/j.eclinm.2023.102143 PMC10432999

[66] Tao W Lagergren J. Clinical management of obese patients with cancer. Nat Rev Clin Oncol 2013;10:519–33. 23856746 10.1038/nrclinonc.2013.120

